# School in Italy: a safe place for children and adolescents

**DOI:** 10.1186/s13052-021-00978-w

**Published:** 2021-02-02

**Authors:** Alberto Villani, Luana Coltella, Stefania Ranno, Federico Bianchi di Castelbianco, Paola Maria Murru, Rossella Sonnino, Teresa Mazzone, Livia Piccioni, Giulia Linardos, Stefano Chiavelli, Fabrizio Pontarelli, Giovanni Corsello, Massimiliano Raponi, Carlo Federico Perno, Carlo Concato

**Affiliations:** 1 Emergency Department General Pediatrics, Bambino Gesù Children Hospital, IRCCS, Rome, Italy; 2 Italian Pediatric Society, Rome, Italy; 3 Microbiology Unit, Bambino Gesù Children Hospital, IRCCS, Rome, Italy; 4 Ortofonologia Institute, Rome, Italy; 5 SS Maria Addolorata School, Rome, Italy; 6 Regina Elena School, Rome, Italy; 7 ASL RM 1, Rome, Italy; 8 Pediatric Department and Intensive Neonatal Unit, Palermo University, Palermo, Italy; 9 Health Management Department, Bambino Gesù Children Hospital, IRCCS, Rome, Italy

**Keywords:** SARS-CoV-2, School, COVID, Prevention measures

## Abstract

**Background:**

During the first SARS-CoV-2 pandemic phase, the sudden closure of schools was one of the main measures to minimize the spread of the virus. In the second phase, several safety procedures were implemented to avoid school closure.

To evaluate if the school is a safe place, students and staff of two school complexes of Rome were monitored to evaluate the efficacy of prevention measures inside the school buildings.

**Methods:**

Oral secretions specimens were collected from 1262 subjects for a total of 3431 samples, collected over a 3 months period.

Detection of Coronavirus SARS-CoV-2 was performed by real-time PCR. Target genes were represented by E gene, RdRP/S gene and N gene.

**Results:**

Among the 3431 samples analyzed, just 16 sample resulted as positive or low positive: 1 sample in the first month, 12 samples in the second month and 3 in the third month.

In each period of evaluation, all positive children attended different classes.

**Conclusions:**

Even if the school has the potential for spreading viruses, our preliminary results show the efficacy of the implementations undertaken in this setting to minimize virus diffusion.

Our evidence suggests that school does not act as an amplifier for transmission of SARS-CoV-2 and can be really considered a safe place for students.

## Background

During the first SARS-CoV-2 pandemic phase, the sudden closure of schools has been perceived as one of the measures to minimize the spread of the virus.

In the second phase, in order to guarantee adequate and appropriate educational and social learning and children development, and ensuring a continuous prevention and minimization of SARS-CoV-2 transmission in the school setting, several safety procedures were implemented [1, 2].

In particular, administrative policies, infrastructural adjustments, sanitation of environments, appropriate use of individual protection devices, symptoms screenings by parents and teachers, have been the major measures undertaken at the school level.

To answer the question if the school is a safe place, students and staff of two school complexes of Rome were monitored thrice (at the beginning of the school year in September, in October and in November), to evaluate the efficacy of prevention measures inside the school buildings.

## Methods

Oral secretions specimens were collected in the buccal cavity by oral swabs and inoculated in 3 ml of Universal Transport Medium (UTM) [3].

A total of 3431 samples were collected at different times, 1099 samples were collected in the period 21st September - 12th October 1075 in the period 19th October - 13th November, and 1257 in the period 16th November - 4th December.

For the detection of the Coronavirus SARS-CoV-2, a molecular test was performed.

In particular, the nucleic acid was extracted using the STARMag Universal Cartridge kit (Seegene) on the automated Starlet platform: a total of 200 μl per sample were extracted and eluted with 100 μl of elution buffer.

Real-time PCR was performed on CFX96 (Bio-Rad Laboratories) with Allplex™ 2019-nCoV Assay. Target genes were represented by E gene, RdRP/S gene and N gene.

For detection were used 5 μl of the extracted RNA, in a final volume of 20 μl. The results were analyzed automatically using Seegene software (Seegene n-Cov Viewer).

Samples were considered negative when no gene was detected, positive when three genes were detected and low positive when one or two genes were detected.

## Results

The study population consisted of 1265 subjects, including 1097 students, 141 teachers and 27 school employees, all coming from two different school complexes.

On the basis of the consent given to the study, following an interview with a pediatrician and a psychologist, subjects who accepted to participate in the study were 1251: 132 out of 132 (100%) from preschool (aged < 6 years), 369 out if 369 (100%) from primary school (between 6 and 10 years), 414 out of 416 (99,52%) from secondary school (between 11 and 13 years), 336 out of 348 (96,55%) from upper secondary school (between 14 and 18 years). Data are summarized in Table 1.

**Table 1:** Summary of subjects included in the study

School level	N° students	N° teachers	N° school employees	Total
Preschool (< 6 years)	119	8	5	132
Primary (6–10 years)	331	30	8	369
Secondary (11–13 years)	347	58	9	414
Upper secondary (14–18 years)	286	45	5	336
Total	1083	141	27	1251

Among the 1251 subject tested, 16 resulted positive: 6 females and 10 males, with a mean age of 18 years (range 5–54 years).

Our results show that, among the 1099 samples analyzed in the first round, just one sample resulted as positive. The sample referred to a student of the upper secondary school.

Among the 1075 samples analyzed in the second round, 7 resulted positive (an adult and 6 students) and 5 low positive (two adults and 3 students).

Among the 1257 samples analyzed in the third round, 3 resulted positive (3 students).

Globally, 16 positive/low positive subjects were distributed in 14 different classrooms: 1 in preschool, 6 in primary school, 3 in secondary school, and 6 in upper secondary school.

Only two classrooms presented more than one positive result, in particular, two students (in different rounds of testing) and a student and an adult sharing a class environment, in the second round.

## Discussion

Evidence from past literature shows that the school has in general the potential for spreading viruses, particularly in case of flu viruses, when only limited precautionary measures are taken [4].

Studies in educational settings conducted during the first coronavirus disease 2019 (COVID) pandemic phase suggest that staff-to-staff transmission was more common than staff to student and student-to-student transmission [5, 6]. Evidence from countries that have reopened or never closed schools suggest that these environments are not associated with a significant increase in community transmission [7].

Our results are still preliminary since the whole project foresees a monthly monitoring during one semester to show the efficacy of the implementations undertaken by the school to minimize virus diffusion. Nevertheless, on a total of 1262 subjects included in the study, only 16 resulted positive/low positive (1.3%) over a period of approximately 3 months, starting from September 24th until the beginning of December 2020.

Since the collection of samples was carried out at the school premises (where symptomatic persons are not allowed to enter school), it is conceivable that none of the positive subjects presented symptoms at the time of diagnosis.

Regarding the two students who resulted positive in the same classroom but in different rounds (collected 1 month apart), we can assume that they are not related, because usually the incubation time of the infection is shorter than 1 month and viral infectivity is high in community contexts [8], and therefore the signs of the infection transmitted by the student would have appeared far before a month.

Among the 12 subjects who were tested positive in the second round, there were a student and an adult who shared the same classroom. It should be noted that: 1. The adult (a Communication Education Assistant of the primary school) participated also to the teaching activities of other classes; 2. None of the students who attended the same class, or the other students who had interacted with this adult, turned to be positive. Therefore, it is reasonable that their concomitant infection was not interrelated.

Among the three students of primary school tested positive in the third round, two were relatives.

Positive results distribution in the three rounds, respectively 0.1, 1.1 and 0.2%, is consistent, and even lower than Epidemiology of SARS-CoV-2 infection in Rome during the months of September–October, October–November and November–December, which indicates, together with the epidemiological analysis, that the infections are detected at school rather than caused by viral transmission at school [9].

## Conclusions

Our evidence suggests that school does not act as an amplifier for transmission of SARS-CoV-2, while other settings where young people congregate may be a greater carrier of the community transmission and setting-linked risk amplifiers [4, 6, 10].

Further procedures implemented in classrooms, including physical distancing, frequent hand hygiene, cleaning and disinfection, adequate ventilation, have been effective in preventing the introduction and spread of SARS-CoV-2 in the educational setting in Italy.

In conclusion, according to the data that emerged from this study, the school in Italy, thanks to all the procedures adopted, can be considered a safe place for children and adolescents.

## Data Availability

The datasets used and/or analyzed during the current study are available from the corresponding author on reasonable request.
